# Subcutaneous Migration in Paragonimiasis as a Diagnostic Clue in an Unusual Presentation: A Case Report

**DOI:** 10.7759/cureus.90032

**Published:** 2025-08-13

**Authors:** Mako Eguchi, Keisuke Masumoto, Mio Kokubo-Tanaka, Haruhiko Maruyama, Satoru Morimoto, Yuichi Mizuta

**Affiliations:** 1 Respiratory Medicine, St. Mary's Hospital, Fukuoka, JPN; 2 Parasitology, Department of Infectious Diseases, Faculty of Medicine, University of Miyazaki, Miyazaki, JPN; 3 Regenerative Medicine, Keio University Regenerative Medicine Research Center, Kanagawa, JPN

**Keywords:** eosinophilia, migration tract, paragonimiasis, praziquantel, pseudochylothorax, subcutaneous mass

## Abstract

Paragonimiasis is a foodborne parasitic infection caused by trematodes of the genus *Paragonimus*, primarily *P. westermani* and *P. miyazakii* in Japan. Though once endemic, its incidence has declined significantly in recent decades. However, globalization, changes in dietary customs, and an increase in foreign-born residents have contributed to the emergence of new cases, particularly in urban settings.

We present a case of a 25-year-old man from Cambodia, living in Japan for two years as a technical intern trainee, who developed a productive cough and pleuritic chest pain. He reported consuming raw Japanese mitten crabs seven months earlier. Initially misdiagnosed with pneumonia, his symptoms persisted despite antibiotic therapy. CT imaging revealed pleural effusion, a cavitary lesion, and a linear tract consistent with worm migration. A subcutaneous abdominal mass was noted and surgically removed. Histopathological examination revealed eosinophilic infiltration and serpiginous necrosis, suggesting larval migration. Laboratory tests showed marked eosinophilia (11,011/μL), and pleural fluid analysis indicated pseudochylothorax. ELISA confirmed antibodies against *P. westermani* and *P. miyazakii*. The patient was treated with praziquantel, leading to complete clinical and radiological resolution.

This case highlights the diagnostic challenges of paragonimiasis, especially with atypical presentations. Subcutaneous lesions are rare and often mimic benign tumors or other parasitic diseases, such as sparganosis or cysticercosis. The pulmonary findings may be confused with tuberculosis or eosinophilic pneumonia. Thus, thorough dietary and travel history-taking, cultural awareness, and a multidisciplinary diagnostic approach, including imaging, histology, and serology, are essential. Diagnosis of paragonimiasis should rely on a combination of clinical presentation, radiological findings, and serological testing, as ectopic manifestations are uncommon and may not be promptly recognized. This case underscores the need for heightened clinical awareness of re-emerging parasitic diseases in increasingly multicultural societies.

## Introduction

Paragonimiasis is a zoonotic trematode infection that humans acquire by ingesting raw or undercooked freshwater crabs or crayfish harboring metacercariae, which are the encysted larval stage of the parasite infectious to humans. In Japan, infections are primarily caused by *Paragonimus westermani* and *Paragonimus miyazakii* [1]. Historically, the disease was highly endemic across Japan, with over 300,000 estimated cases in the late 1950s [2]. Intensive public health interventions during the 1950s and 1960s, including mass screenings and education campaigns, drastically reduced new infections, and by the 1970s, paragonimiasis was considered a disease of the past [3,4]. However, since the late 1980s, re-emergent cases have been documented [3], and from 2001 to 2012, a nationwide review identified 443 confirmed cases of paragonimiasis in Japan, of which 113 (25.5%) were in immigrant patients, while the remaining 330 cases (74.5%) occurred in native Japanese individuals [2]. Current reports average fewer than 50 cases annually [1].

Despite this reduction, paragonimiasis has not disappeared. The increasing diversity of dietary habits, rise in international migration, and importation of exotic or traditional foods have contributed to the disease’s persistence and even re-emergence in non-endemic areas. Among the aforementioned 443 patients, asymptomatic cases were also reported, suggesting that detection was not solely symptom-driven. Although the review did not detail the screening methods, diagnoses appear to have relied on a combination of clinical, radiological, and serological findings [2]. In metropolitan areas such as Tokyo and Osaka, immigrant populations now represent the majority of patients, reflecting not only differences in food practices but also diagnostic gaps in urban medical settings unfamiliar with parasitic diseases.

Clinically, paragonimiasis is known for its varied presentation. Pulmonary involvement typically presents with productive cough, chest pain, or hemoptysis - symptoms that closely resemble tuberculosis. Ectopic forms, however, may involve the brain, liver, peritoneum, or subcutaneous tissues. Among these, subcutaneous migration is particularly rare, occurring in less than 5% of cases according to a 12-year retrospective study in Japan [5]. Subcutaneous nodules are often misinterpreted as benign tumors or other parasitic infections such as sparganosis or cysticercosis, which can delay diagnosis and treatment.

Early diagnosis of paragonimiasis relies on a high index of suspicion, especially in patients presenting with eosinophilia and a relevant dietary history. While identification of eggs in sputum or stool remains the traditional diagnostic method, serological tests are increasingly essential in atypical or extrapulmonary cases. Moreover, histological examination of subcutaneous lesions may reveal eosinophilic necrosis, a valuable but underappreciated clue of larval migration.

This report presents a unique case of pulmonary paragonimiasis with subcutaneous involvement in an immigrant patient. The sequence of clinical events - from dietary exposure to subcutaneous nodule formation and pulmonary disease - offers rare insight into the parasite's migratory pathway. This case emphasizes the importance of integrating epidemiological, radiological, pathological, and immunological data for accurate diagnosis. In an era of global mobility, awareness of such re-emerging infections remains critical, even in countries where the disease is no longer endemic.

## Case presentation

We report a case of a 25-year-old man from Cambodia, who had been living in Japan for two years as a technical intern trainee. He presented with a three-month history of productive cough and pleuritic chest pain. He had consumed raw Japanese mitten crabs seven months earlier. He had no other freshwater-related activities apart from crab consumption. Two months after ingestion, he developed transient abdominal pain, followed by progressive respiratory symptoms. He was initially diagnosed with pneumonia at a local clinic and treated with antibiotics without improvement. He was subsequently referred to our hospital.

**Figure 1:**
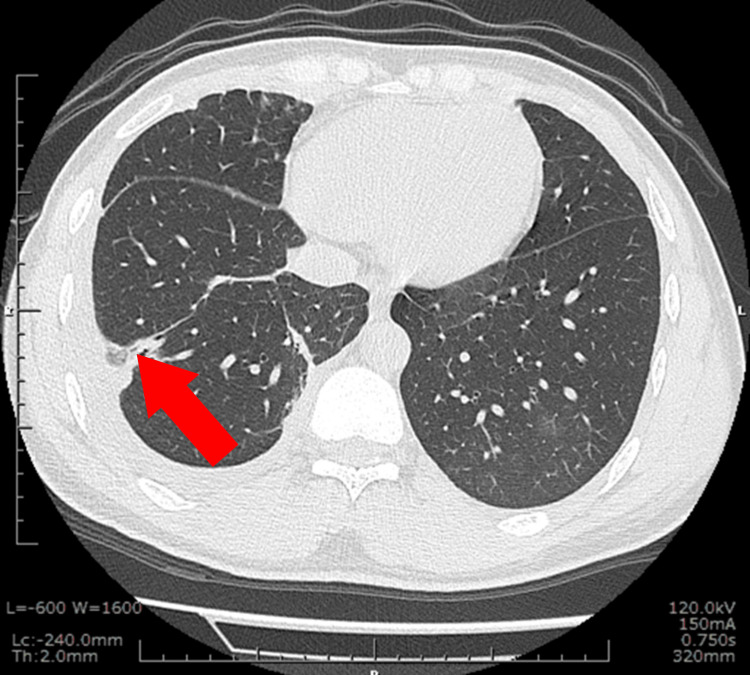
Non-contrast chest CT image Non-contrast chest CT shows an infiltrative shadow in the right lower lobe, with a cavity-like lesion suggestive of a migration tract. The red arrow indicates a subpleural linear opacity abutting the visceral pleura, a finding suggestive but not pathognomonic of paragonimiasis.

On presentation, he was afebrile and hemodynamically stable. Physical examination revealed a 4-cm, soft, mobile, non-tender, and rubbery subcutaneous mass in the right lower abdomen without overlying erythema. He denied any history of trauma or insect bites. Laboratory testing revealed marked peripheral eosinophilia (11,011/μL) (Table 1). Chest computed tomography (CT) showed right-sided pleural effusion, a small cavitary lesion, and ground-glass opacity in the left lower lobe, and a linear tract extending toward the pleura, consistent with a worm migration tract (Figure 1).

**Table 1:** Blood Test Results Summary

Laboratory parameter	Value	Reference range
White Blood Cell Count	18.2×10³/µL	3.5-9,0×10³/µL
Band neurtophils	0.0%	0-5%
Segmented neutrophils	24.0%	40-70%
Monocytes	0.5%	2-8%
Lymphocytes	14.0%	20-45%
Basophils	1.0%	0-2%
Eosinophils	60.5%	0-6%
Red Blood Cell Count	4.79×10^6^/µL	Males: 4.3-5.3×10^6^/µL
Platelet count	277×10^3^/µL	130-350×10^3^/µL
Aspartate Aminotransferase (AST)	35 U/L	13-30 U/L
Alanine Aminotransferase (ALT)	62 U/L	Males: 10-42 U/L
Gamma-Glutamyl Transpeptidase (γ-GTP)	32 U/L	13-64 U/L
Creatine Kinase (CK)	59 U/L	Males: 10-42 U/L
Amylase	58 U/L	Males: 59-248 U/L
Total protein	9.9 g/dL	6.6-8.1 g/dL
Sodium	141.4 mmol/L	138-145 mmol/L
Potassium	5.41 mmol/L	3.6-5.1 mmol/L
Chloride	103.3 mmol/L	96-106 mmol/L
C-Reactive Protein (CRP)	0.76 mg/dL	<0.3 mg/dL

Pleural fluid analysis demonstrated exudative features (protein 9,080 mg/dL, lactate dehydrogenase (LDH) 4,512 IU/L) with a white blood cell count of 3,093/μL, 83.2% of which were eosinophils. Cholesterol and triglyceride levels were 379 mg/dL and 47 mg/dL, respectively, suggesting pseudochylothorax (Table 2). Serological testing using an ELISA (enzyme-linked immunosorbent assay) was positive for antibodies against both *P. westermani* and *P. miyazakii*, confirming the diagnosis of paragonimiasis.

**Table 2:** Pleural Effusion Test Results Summary The pleural effusion was classified as exudative based on Light’s criteria, with elevated protein and Lactate dehydrogenase (LDH) levels.

Pleural Fluid Analysis parameter	Value
Total cell count	3,093/µL
Neutrophils	0.2%
Lymphocytes	15.6%
Eosinophils	83.2%
Total protein	9,080 mg/dL
Glucose	3 mg/dL
Lactate dehydrogenase	4,512 IU/L
Triglycerides	47 mg/dL
Total cholesterol	379 mg/dL

## Discussion

Pathophysiology and clinical manifestations

**Figure 2:**
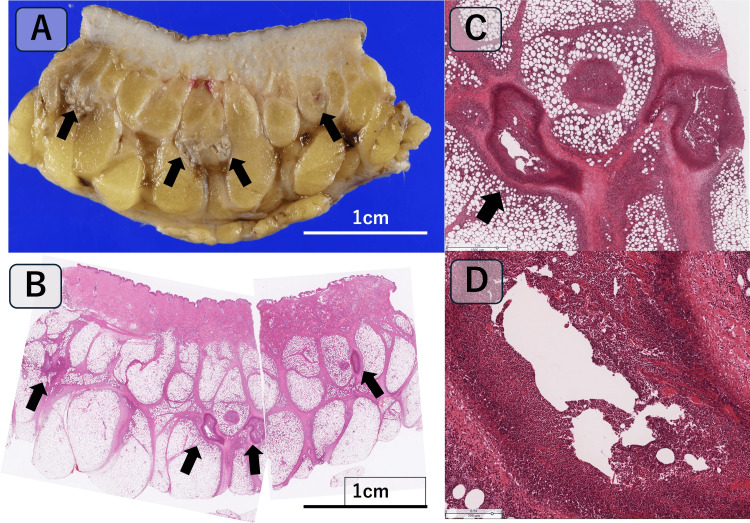
Gross and Histopathological Appearance of the Surgically Resected Subcutaneous Mass and Lung Lesions Associated With Paragonimus Infection. (A) Gross appearance of the subcutaneous mass: The mass primarily consists of adipose tissue without identifiable parasitic eggs or worm bodies. Black arrows indicate whitish fibrotic areas within the adipose tissue, which correspond to parasite-induced lesions. (B) Histopathological examination of the subcutaneous mass: Hematoxylin and eosin (H&E) staining at low magnification reveals prominent eosinophilic infiltration and a serpiginous necrotic tract approximately 1 mm in width, suggestive of larval migration. (C) Histological appearance of lung lesions: Low-power view (H&E stain, original magnification ×1.5) showing a pale, serpiginous necrotic area indicated by a black arrow, consistent with coagulative necrosis associated with *Paragonimus* infection. (D) High-power view of lung lesions: High-power view (H&E stain, original magnification ×6.5) corresponding to the necrotic area shown in (C). The necrotic tract is irregularly shaped, pale, and structureless, with preserved tissue architecture but loss of nuclei. Dense inflammatory cell infiltration is observed around the necrotic area, reflecting tissue damage along the parasite’s migration pathway.

During the migratory phase, larvae penetrate the intestinal wall, causing abdominal pain, and later migrate to the pleural cavity, resulting in respiratory symptoms [1,4]. Histopathological examination of the surgically resected subcutaneous mass revealed serpiginous necrosis approximately 1 mm in width with prominent eosinophilic infiltration (Figure 2). Although no eggs or adult worms were identified, these findings strongly suggested ectopic subcutaneous migration of *Paragonimus* larva.

The life cycle of *Paragonimus* involves a complex multi-host system. After ingestion, metacercariae excyst in the small intestine, and the released larvae penetrate the intestinal wall, entering the peritoneal cavity. The larvae may then migrate to the abdominal wall muscles before traversing the diaphragm to reach the pleural cavity and eventually the lung parenchyma, where they mature into adult worms within fibrous cysts [6]. However, aberrant migration can occur, leading to ectopic paragonimiasis affecting various organs, including the brain, liver, and subcutaneous tissues [1,5,7].

The pseudochylothorax observed in this case represents a distinctive but infrequently reported manifestation of pleural paragonimiasis. Unlike typical chylous effusions, pseudochylothorax is characterized by high cholesterol content without elevated triglycerides, often developing in long-standing inflammatory pleural diseases [8]. The chronic inflammatory response to a migrating larva likely contributed to the development of this finding in our patient.

Diagnostic considerations

In paragonimiasis, cerebral and subcutaneous lesions are among the more commonly reported extrapulmonary manifestations. However, subcutaneous involvement remains relatively rare in clinical practice. In a 12-year retrospective study in Japan that analyzed 384 reported cases of paragonimiasis regardless of nationality, subcutaneous lesions were identified in only 4.9% of patients [1,5]. These nodules are typically painless, mobile, and may mimic benign soft tissue tumors or other parasitic infections such as sparganosis, leading to diagnostic confusion. Even in the absence of parasite identification, histological evidence of eosinophilic necrosis can serve as an indirect but valuable clue of larval migration.

Diagnostic approaches for paragonimiasis should integrate multiple methodologies. While traditional diagnosis relies on identification of *Paragonimus* eggs in sputum or stool samples, serological testing has become increasingly important, particularly for cases with ectopic manifestations where egg detection may be challenging [1,2]. Immigrant patients from endemic regions typically present with more advanced disease, multiple lung lesions, and a higher likelihood of ectopic manifestations compared to native Japanese patients [3,4]. Previous epidemiological analyses have shown that subcutaneous nodules and other ectopic manifestations are more frequently observed in immigrant patients from endemic regions, whereas Japanese patients more commonly present with typical pulmonary symptoms such as cough, sputum, and chest pain [1]. This difference in clinical presentation underscores the need for heightened awareness of ectopic paragonimiasis in immigrant populations.

Our patient exhibited the characteristic marked eosinophilia (present in approximately 80% of immigrant cases) that should prompt consideration of parasitic infection in the appropriate clinical context. The patient, originally from Cambodia, where *P. westermani* is known to be present, may have acquired the infection before migrating. However, given the timing of symptom onset and the patient’s history of consuming raw freshwater crabs (*Eriocheir japonica*) in Japan, domestic acquisition is considered more likely. In Cambodia, consumption of raw or undercooked freshwater crabs is a traditional practice in certain rural regions. If such dietary habits are maintained after migration to Japan, particularly when using locally caught species such as *Eriocheir japonica*, the potential for domestic transmission persists. This cultural-ecological link illustrates how transmission can occur even in non-endemic settings. Although certain radiological findings, such as linear tracts abutting the visceral pleura, suggest paragonimiasis, they are not pathognomonic. For example, hydatid disease caused by *Echinococcus granulosus* typically presents with well-defined cystic lesions [9]. Neither of these findings should be interpreted in isolation; integration with clinical history, eosinophilia, and serological evidence is crucial for an accurate diagnosis.

In contrast to previous reports, this case clearly demonstrated a chronological sequence: ingestion of raw crab, early abdominal pain, development of a subcutaneous nodule, and subsequent pulmonary lesions. Although some prior cases have documented adult worms or eosinophilic granulomas in subcutaneous tissue, reports demonstrating direct pathological correlation with the worm migration tract, as seen in this case, are rare. The typical migratory route of *Paragonimus* larvae from the intestine to the lungs via the peritoneal cavity and diaphragm has been well documented in parasitological studies using animal models [10]. Although the exact mechanism by which the larvae enter subcutaneous tissue remains unclear, the pathological findings in this case, specifically, the identification of a migration tract within the subcutaneous lesion, provide strong supporting evidence for the possibility of subcutaneous ectopic migration during this established migratory process.

Treatment and outcomes

**Figure 3:**
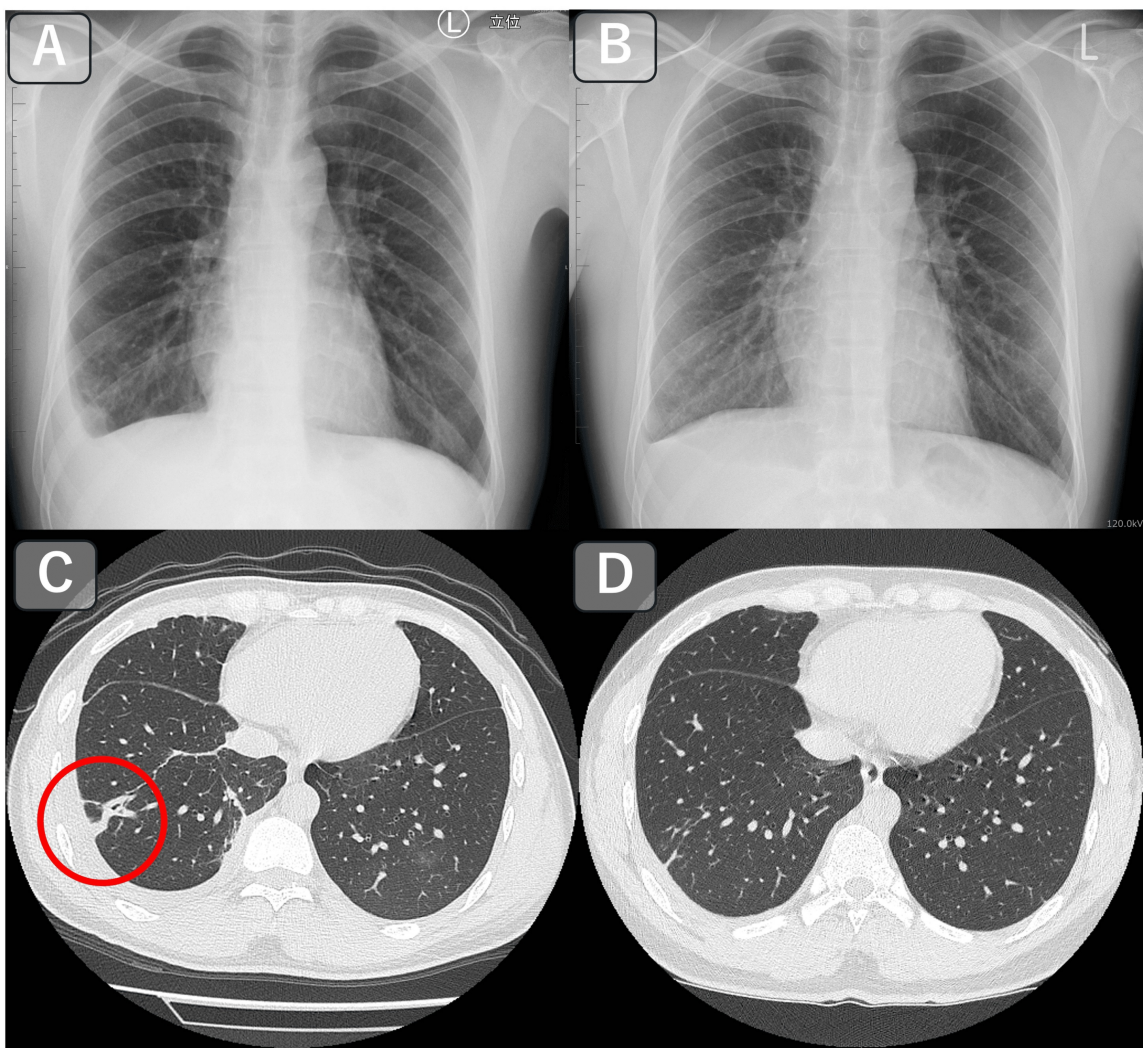
Radiographic and CT Findings Before and After Treatment. (A) Chest X-ray prior to treatment showing right-sided pleural effusion. (B) Chest X-ray taken three months after praziquantel treatment, demonstrating resolution of the pleural effusion. (C) Chest CT prior to treatment showing a linear opacity with a cavitary lesion in the right lung (circled). (D) Chest CT three months after treatment showing resolution of the previously noted cavitary lesion and linear opacity.

This case underscores the importance of a comprehensive diagnostic approach that integrates dietary history, imaging findings, serological tests, and histopathological evaluation in paragonimiasis. The patient was treated with oral praziquantel (3 × 25.0 mg/kg for three consecutive days). This treatment regimen is also described in the handbook on parasitic diseases published by the Japan Agency for Medical Research and Development (AMED), and is widely referenced in clinical practice in Japan. At three-month follow-up, the peripheral eosinophil count had decreased to 607/μL, the pleural effusion had resolved, and CT findings had improved (Figure 3). The subcutaneous mass did not recur, and chest pain gradually subsided. At final follow-up, the patient remained asymptomatic.

Clinical trials of praziquantel for paragonimiasis have demonstrated excellent efficacy [11,12]. One study showed that administration of praziquantel at 3 × 25.0 mg/kg for three consecutive days achieved a 100% cure rate at four-month follow-up examinations [12]. The medication is generally well-tolerated, with mild and transient side effects such as headache and dizziness. Resolution of radiographic abnormalities and disappearance of serological markers provide additional confirmation of successful treatment.

## Conclusions

This case underscores the importance of considering parasitic infections when eosinophilia is present, particularly in combination with eosinophilic pleural effusion and cavitary lung lesions. Although rare, ectopic manifestations such as subcutaneous migration can serve as important diagnostic clues and broaden our understanding of the diverse clinical presentations of paragonimiasis. Clinicians should maintain heightened awareness of this condition, particularly in immigrant populations from endemic areas or individuals with relevant dietary exposures. Specific dietary history questions, such as "Have you eaten raw or undercooked freshwater crabs?" can help identify potential sources of infection. The case illustrates the value of integrating clinical, radiological, serological, and histopathological findings to achieve accurate diagnosis and implement appropriate therapy for this increasingly uncommon but still relevant parasitic infection. Regardless of location, public health education should emphasize the avoidance of raw or undercooked freshwater crabs, including imported products, to prevent paragonimiasis.
